# Inhibitor of apoptosis protein expression in glioblastomas and their *in vitro* and *in vivo* targeting by SMAC mimetic GDC-0152

**DOI:** 10.1038/cddis.2016.214

**Published:** 2016-08-04

**Authors:** A Tchoghandjian, A Soubéran, E Tabouret, C Colin, E Denicolaï, C Jiguet-Jiglaire, A El-Battari, C Villard, N Baeza-Kallee, D Figarella-Branger

**Affiliations:** 1 Aix-Marseille University, Inserm, CRO2 UMR_S 911, Marseille, France; 2 AP-HM, Timone Hospital, Department of Neuro-Oncology, Marseille, France; 3 AP-HM, Timone Hospital, Department of Anatomopathology, Marseille, France

## Abstract

Glioblastomas (GBMs) are the most aggressive primary brain tumors in adult and remain a therapeutic challenge. Targeting key apoptosis regulators with the ultimate aim to restore apoptosis in tumor cells could be an interesting therapeutic strategy. The inhibitors of apoptosis proteins (IAPs) are regulators of cell death and represent attractive targets, especially because they can be antagonized by SMAC mimetics. In this study, we first investigated the expression of cIAP1, cIAP2, XIAP and ML-IAP in human GBM samples and in four different cell lines. We showed that all GBM samples and GBM cell lines expressed all these IAPs, although the expression of each IAP varied from one case to another. We then showed that high level of ML-IAP predicted worse progression-free survival and overall survival in both univariate and multivariate analyses in two independent cohorts of 58 and 43 primary human GBMs. We then used GDC-0152, a SMAC mimetic that antagonizes these IAPs and confirmed that GDC-0152 treatment *in vitro* decreased IAPs in all the cell lines studied. It affected cell line viability and triggered apoptosis, although the effect was higher in U87MG and GL261 than in GBM6 and GBM9 cell lines. *In vivo*, GDC-0152 effect on U87MG orthotopic xenografts was dose dependent; it postponed tumor formation and slowed down tumor growth, significantly improving survival of GBM-bearing mice. This study revealed for the first time that ML-IAP protein expression correlates with GBM patient survival and that its antagonist GDC-0152 improves outcome in xenografted mouse.

Glioblastomas (GBMs), the most common primary brain tumors in adult, remain a therapeutic challenge. Classified as grade IV by WHO, the aggressiveness of GBMs mainly resides in their highly invasive and proliferative behavior, and in their cellular and molecular heterogeneity.^1^ Despite the many clinical trials ongoing, few therapeutic improvements have been made in these past 10 years, with the median overall survival (OS) remaining at ∼15 months. The standard of care is still currently unchanged since 2005 and consists of combining radiotherapy with concomitant and adjuvant temozolomide.^2^ Recently, a promising anti-angiogenic therapy (Bevacizumab) failed to improve OS in two independent phase III trials (AVAglio and RTOG 0825), supporting evidence that GBMs are refractory to treatments.^3, 4^

Treatment resistance is a hallmark of cancer cells that develop strategies to bypass cell death.^5^ Inhibitor of apoptosis proteins (IAPs) are a well-conserved family of eight proteins including cIAP1 (cellular inhibitor of apoptosis protein-1), cIAP2 (cellular inhibitor of apoptosis protein-2), XIAP (X-linked inhibitor of apoptosis protein) and ML-IAP (melanoma inhibitor of apoptosis protein), often expressed in many human cancers.^6^ IAPs are anti-apoptotic proteins contributing to treatment resistance by inhibiting caspase activation. Therefore, their expression in cancers is usually correlated with poor prognosis.^7, 8^ IAPs are characterized by the presence of one to three *Baculovirus IAP Repeat* (BIR) domains, necessary for protein–protein interactions like caspase inhibitory interactions, typically arranged in the protein's amino terminus. cIAP1, cIAP2, XIAP and ML-IAP also contain a carboxyl-terminus RING (Really Interesting New Gene) domain, working as an E3-ubiquitin ligase.^9^ Therefore, these IAPs have the capability of auto- or hetero-ubiquitination leading to proteasomal degradation. Thanks to their E3-ubiquitin ligase activity, the level of IAPs can be controlled by endogenous antagonists including SMAC (second mitochondria-derived activator of caspase) released by the mitochondria during the apoptosis process. Small-molecule SMAC mimetics that mimic the N-terminal part of the endogenous SMAC have been designed to antagonize IAPs and are currently under clinical considerations in some solid cancers (e.g., myeloma and ovarian cancers) and in lymphomas (ClinicalTrials.gov).^10^

For patients with GBM, the prognostic value of cIAP1, cIAP2, XIAP and ML-IAP expression remains to be determined.^7, 11^ Therefore, in this study we investigated *ex vivo* the correlation between their expression levels and patient's survival in two retrospective cohorts in order to highlight the most interesting druggable targets. In order to assess the effect of SMAC mimetic on GBM tumors, we then tested *in vitro* the effect of GDC-0152 on four GBM cell lines. Finally, *in vivo* we treated GBM-bearing mice with GDC-0152 and determined the benefit of the treatment on survival and tumor growth.

## Results

### Correlation of IAP protein expression and GBM survival

#### Cohort 1

**Figure 1:**
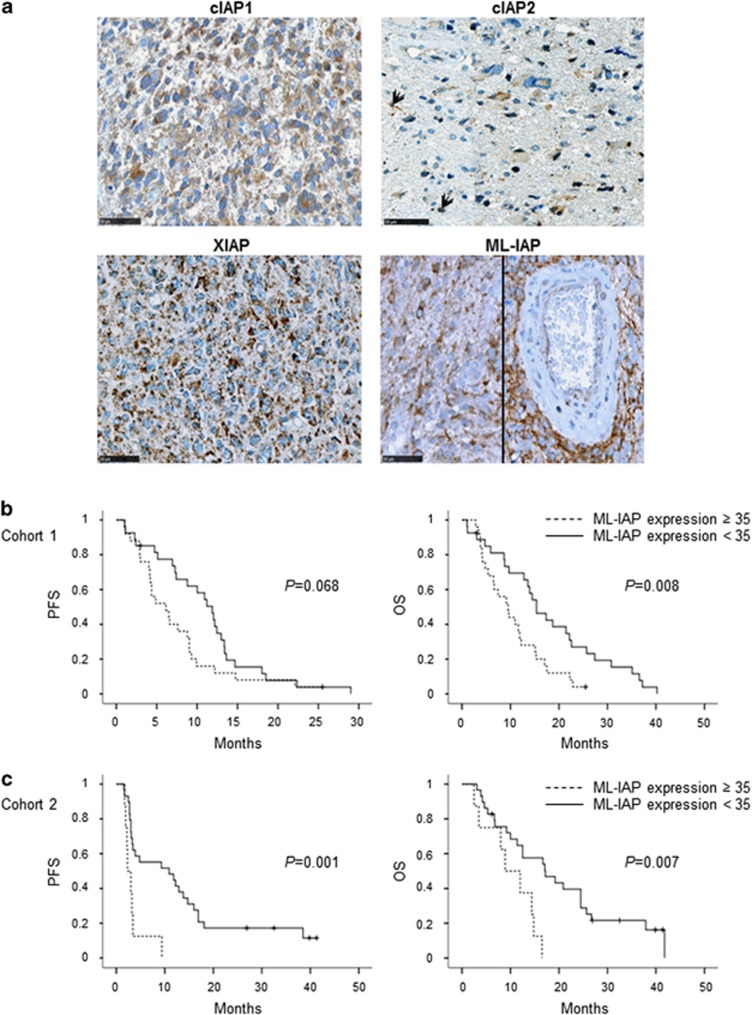
Prognostic value of cIAP1, cIAP2, XIAP and ML-IAP protein expression in human glioblastomas (cohorts 1 and 2). (**a**) cIAP1-, cIAP2-, XIAP- and ML-IAP-positive stainings in GBM. IAPs were heterogeneously expressed by tumor cells in GBM samples (Table 1). Stainings were diffused with a stronger punctuated positivity into the cytoplasm. Black arrows highlight cIAP2-positive nuclei. Scale bars, 50 *μ*m. (**b**) Correlation of ML-IAP protein expression with PFS and OS in cohort 1. The cutoff was 35% and was determined by performing a ROC curve. ML-IAP expression of ⩾35% was correlated with a poor prognosis. (**c**) Correlation of ML-IAP protein expression with PFS and OS in cohort 2. The cutoff was the same as that for cohort 1 analysis (35%). ML-IAP expression of ⩾35% was correlated with a poor prognosis

cIAP1, cIAP2, XIAP and ML-IAP protein expression was first analyzed in a local monocentric cohort of 58 primary GBMs (Table 1). IAPs were heterogeneously expressed by tumor cells in GBM samples (Table 1). Stainings were diffused with a stronger punctuated positivity into the cytoplasm. cIAP2 staining was sparser and was also found into some nuclei. Perivascular organization of ML-IAP-positive cells was often observed (Figure 1a).

As GBM samples expressed IAPs, we then tested for their prognostic values. The majority of samples expressing cIAP1, cIAP2 and XIAP did not exceed 30% of positive cells. Therefore, we analyzed their expression as positive or negative stainings for statistical relevance. In comparison, ML-IAP expression level was homogeneously distributed, allowing us to analyze its expression as a continuous variable. As continuous variable, ML-IAP protein expression was significantly correlated to OS by Cox analysis (*P*=0.005): a smaller expression level was associated with a longer patient OS. Then, receiver operating characteristic (ROC) curve analysis was performed in order to determine an optimal cutoff for next univariate survival analyses. This ROC analysis allowed the determination of ML-IAP optimal cutoff of 35% of cell expression regarding OS (*P*=0.014, AUC: 0.7) (Supplementary Figure S1a). Regarding cIAP1, cIAP2 and XIAP studied as qualitative variables, they were dichotomized into positive and negative immunostainings. In univariate analyses, expression of ML-IAP of ⩾35% was associated with worse prognosis (progression-free survival (PFS): *P*=0.068; OS: *P*=0.008; Figure 1b) and positive expression of XIAP (PFS: *P*=0.057; OS: *P*=0.034; Supplementary Figure S1b) was also associated with worse survival. In multivariate analysis (adjusted by Karnofsky Performance Status, gender and type of surgery), these results remained significant for OS (XIAP: *P*=0.007, hazard ratio (HR): 2.344 (1.261–4.358) and ML-IAP: *P*=0.003, HR: 2.733 (1.415–5.279)). No prognostic value was found for cIAP1 and cIAP2 (Supplementary Figure S2).

#### Cohort 2

In order to validate our previous results, we identified a second independent local cohort of 43 GBMs (Table 1). In this cohort, only ML-IAP expression was correlated with survival in univariate (PFS, *P*=0.001 and OS, *P*=0.007; Figure 1c) and multivariate analyses, adjusted by age and type of surgery (PFS: *P*=0.012, HR: 3.585 (1.320–9.738) and OS: *P*=0.027, HR: 3.09 (1.139–8.385); Supplementary Figure S2) confirming that ML-IAP protein expression is a significant factor of a poor prognosis in GBM. Of note, the pooled analysis of both cohorts 1 and 2 found similar results (Supplementary Figures S3a and b).

### lAP protein expression in GBM cell lines

**Figure 2:**
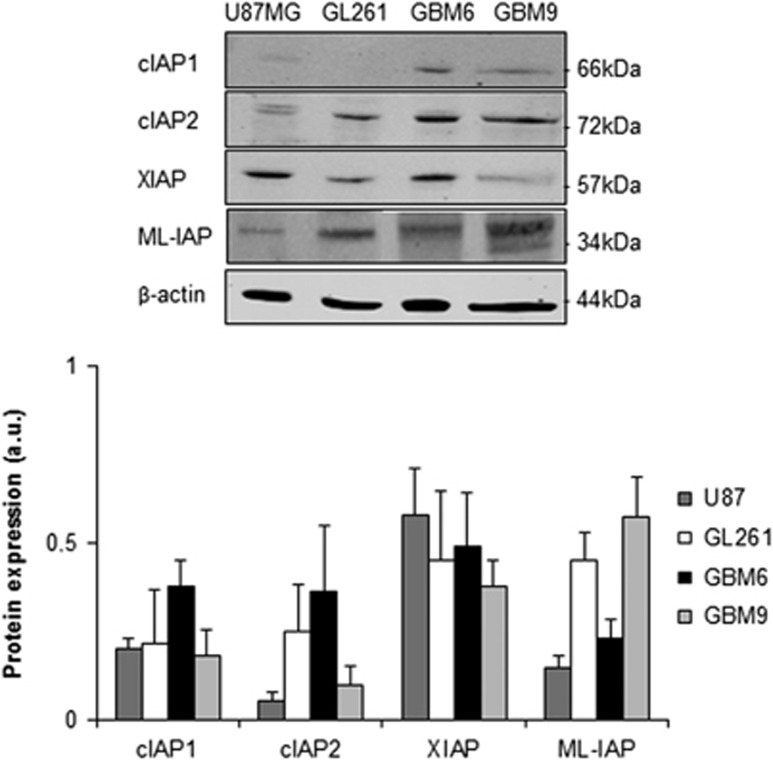
IAP expression in glioblastoma cell lines. Expression levels of cIAP1, cIAP2, XIAP and ML-IAP were analyzed by western blotting and quantified in U87MG and GL261 adherent GBM cell lines, and in GBM6 and GBM9 spheres. Expression level of *β*-actin served as loading control. The four GBM cell lines expressed heterogeneously cIAP1, cIAP2, XIAP and ML-IAP. A representative experiment of four experiments is shown. Quantification was performed using ImageJ software (National Institutes of Health, Bethesda, MD, USA) and data presented were normalized to *β*-actin expression

In order to determine whether we could detect the four IAPs in GBM cell lines, we analyzed their protein levels in U87MG, GL261, GBM6 and GBM9. The four GBM cell lines expressed IAPs at different levels (Figure 2). U87MG cells preferentially expressed XIAP, GL261 cells XIAP and ML-IAP, GBM6 cIAP1, cIAP2 and XIAP, and GBM9 XIAP and ML-IAP.

These results showed that these GBM cell lines expressed the four IAPs including ML-IAP and could therefore be used for further analyses.

### Effect of SMAC mimetic GDC-0152 on cell viability and IAP protein expression in GBM cells

As we found that cIAP1, cIAP2, XIAP and ML-IAP were expressed in GBM and that ML-IAP was an independent factor of bad prognosis in patients, we tested a IAP antagonist targeting the four proteins. We selected a monovalent SMAC mimetic GDC-0152 described to antagonize cIAP1, cIAP2, XIAP and, above all, ML-IAP. Moreover this compound was described to be suitable for both *in vitro* and *in vivo* studies with low toxicity.^10^

**Figure 3:**
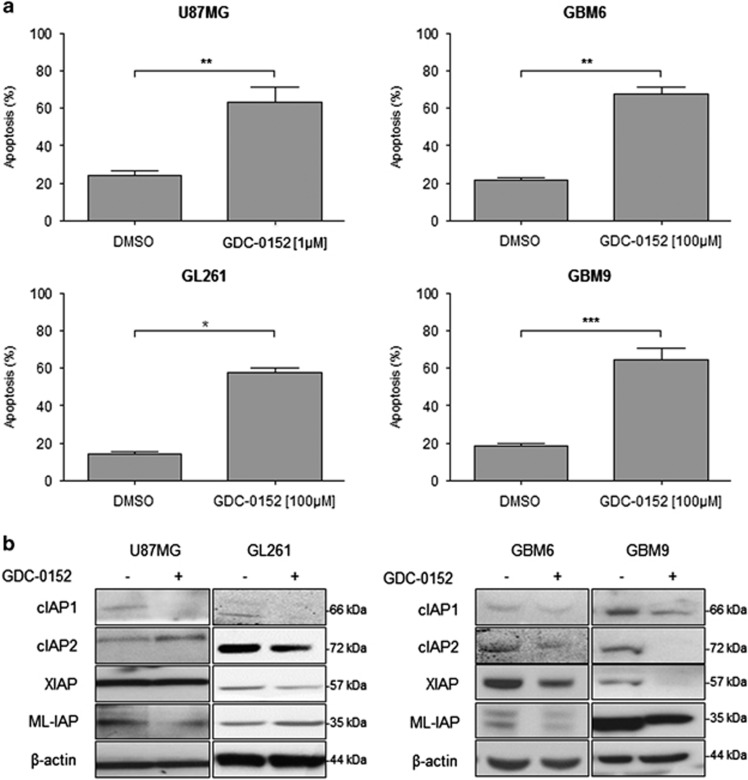
Apoptosis and IAP expression upon SMAC mimetic GDC-0152 treatment in glioblastoma cell lines. (**a**) Apoptosis (SubG0/G1) of DMSO control and GDC-0152-treated cells was determined by flow cytometry of propidium iodide-stained nuclei and percentage of apoptosis is shown. U87MG and GL261 cell lines were treated for 72 h and GBM6 and GBM9 cell lines were treated for 8 days at the indicated concentrations. At these respective time points, percentage of U87MG cells dead by apoptosis, percentage of GL261 cells, percentage of GBM6 cells and percentage of GBM9 cells. Data are expressed as mean+S.E.M. Three independent experiments were performed for the GL261 cell lines and five for the U87MG, GBM6 and GBM9 cell lines. *P<0.05; **P<0.01; ***P<0.005. (**b**) Expression levels of cIAP1, cIAP2, XIAP and ML-IAP were analyzed by western blotting. Cell lines were treated with 1 *μ*M of GDC-0152. U87MG and GL261 were treated for 72 h and GBM6 and GBM9 cell lines for 8 days. In all GBM cell lines GDC-0152 decreased IAP expression. Expression level of *β*-actin served as loading control. A representative experiment of three experiments is shown

GDC-0152 affected U87MG and GL261 cell viability in time- and dose-dependent manner. We can notice that after 24 h of treatment GDC-0152 first increased survival in both cell lines. Cell viability started to decrease at 0.01 *μ*M for U87MG and at 0.5 *μ*M for GL261 after 72 h of treatment. In the same conditions, viability of GBM6 and GBM9 cell lines was barely affected (Supplementary Figure S4). After 72 h of treatment, GDC-0152 triggered 50% of apoptosis in U87MG (*P*=0.0079) and GL261 (*P*=0.05) cell lines at 1 *μ*M and 100 *μ*M, respectively. Eight days of treatment at 100 *μ*M of GDC-0152 were needed to reach 50% of apoptosis in GBM6 (*P*=0.0079) and GBM9 (*P*=0.0022) cell lines (Figure 3a). At these respective time points, GDC-0152 treatment decreased cIAP1 and ML-IAP protein expression in U87MG cell line, cIAP1, cIAP2 and XIAP in GL261 cell line and all IAP expression in GBM6 and GBM9 cell lines (Figure 3b).

Taken together, these results showed that GBM cell lines are sensitive to GDC-0152 treatment and that GDC-0152 is able to affect and decrease all IAP protein expression.

### Effect of SMAC mimetic GDC-0152 on survival and tumor growth *in vivo*

**Figure 4:**
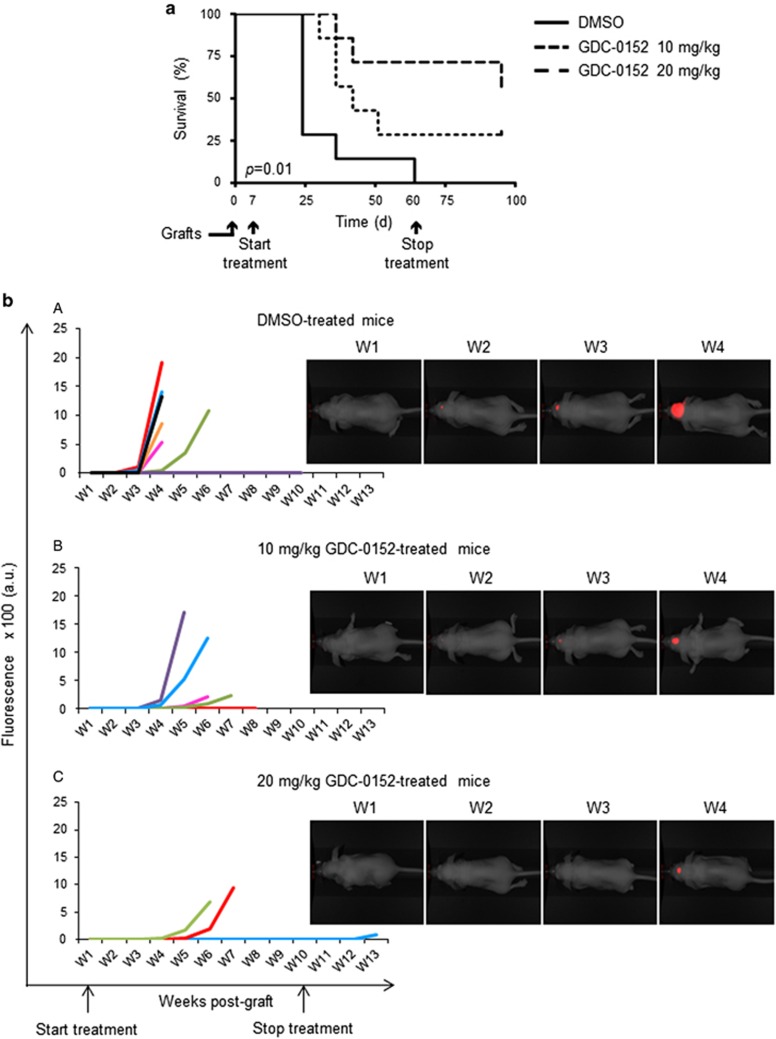
Effect of SMAC mimetic GDC-0152 on survival and tumor growth of mice bearing intracranial tumors. (**a**) A total of 100 000 U87MG-iRFP cells were injected into the corpus callosum of athymic *nude* mice. At 1 week after injection, mice were treated either with DMSO (*n*=7) or 10 mg/kg (*n*=7) or 20 mg/kg (*n*=7) of GDC-0152. OS curves of mice were estimated by the Kaplan–Meier method. GDC-0152 increased mice survival in a dose-dependent manner. (**b**) Images of representative U87MG-iRFP tumors according to treatments are shown. For each group of mice ((A) DMSO group (group A); (B) mice treated with 10 mg/kg GDC-0152 (group B) and (C) mice treated with 20 mg/kg GDC-0152 (group C)), graphs represent the normalized growth curve for each animal that developed a tumor. In addition, we observed that 7/7 mice developed a tumor in group A, 5/7 in group B and 3/7 in group C. In this group one mouse developed a tumor 2 weeks after the end of treatment (blue line). The mouse representative of the DMSO group corresponds to the black line; the mouse representative of the 10 mg/kg GDC-0152 corresponds to the blue line; the mouse representative of the 20 mg/kg GDC-0152 corresponds to the green line. W, weeks. GDC-0152 treatment slowed down tumor growth

**Figure 5:**
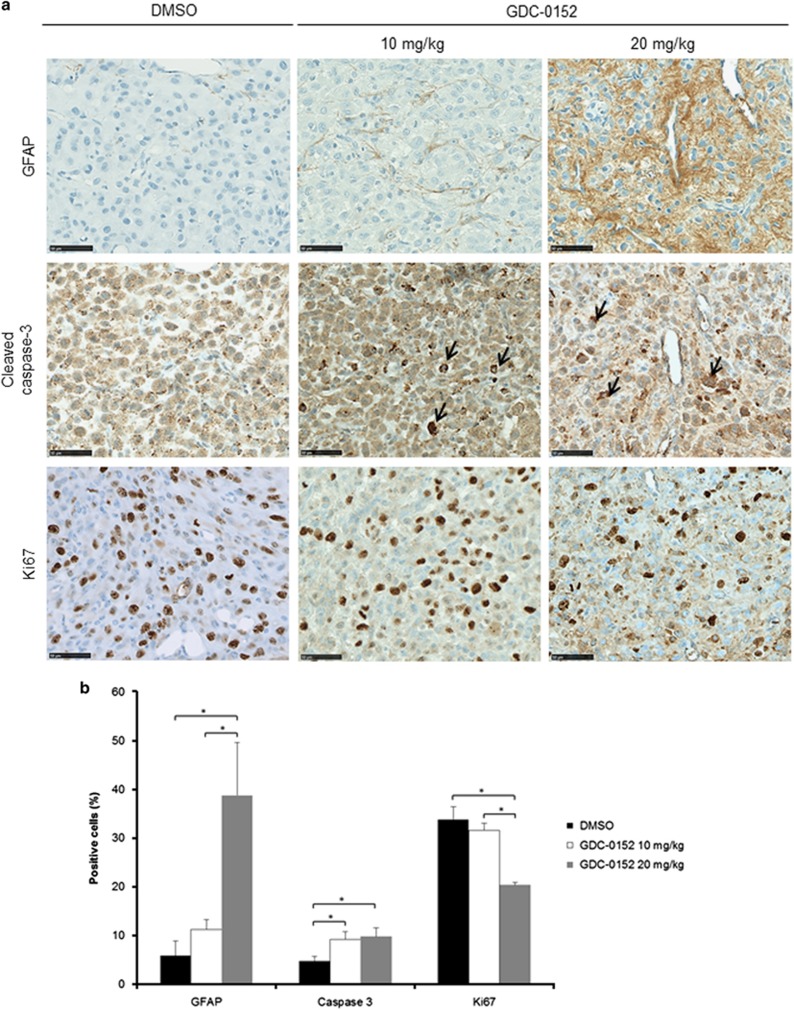
Immunohistochemistry analysis of mice-treated brains. (**a**) Representative GFAP, cleaved caspase-3 and Ki67 stainings of tumors treated with DMSO and 10 and 20 mg/kg of GDC-0152. Black arrows highlight apoptotic cells. Scale bars, 50 *μ*m. (**b**) Quantification of the percentage of GFAP-, cleaved-caspase-3- and Ki67-positive cells in tumors treated with DMSO and 10 and 20 mg/kg of GDC-0152. Differentiation and cell death showed respectively by GFAP and cleaved caspase-3 stainings were increased in GDC-0152-treated brains. Cell proliferation was reduced in GDC-0152-treated tumors as shown by a decrease in Ki67 staining. Mean+S.E.M. are shown. *P<0.05

**Figure 6:**
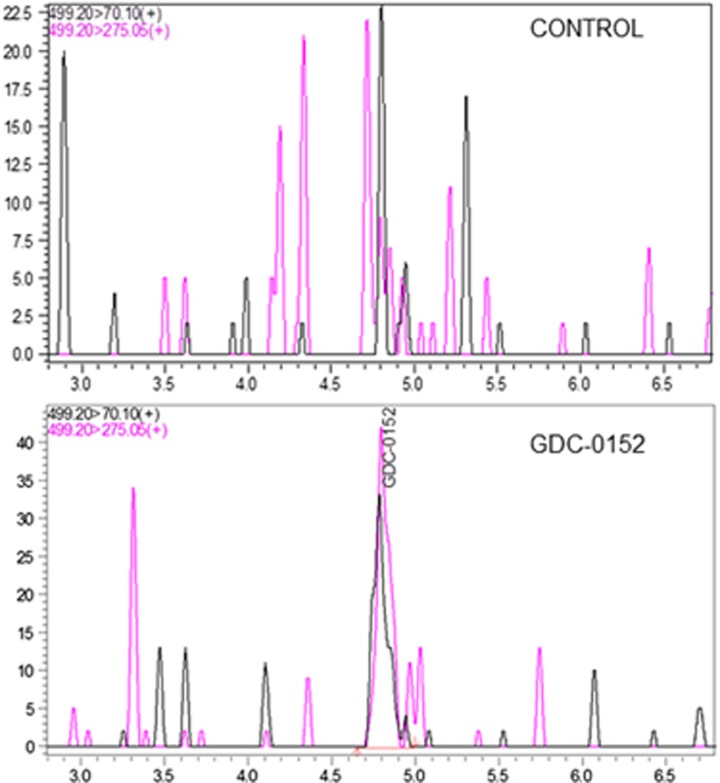
Multiple reaction monitoring (MRM) chromatograms of FFPE tissue extracts from control mouse brain (upper) and GDC-0152-treated mouse brain (lower). Linearity: Standard curve was obtained by linear regression using external calibration with 5 points in the range of 1 to 100 ng/ml. The calibration curve was linear over the range of 1 to 100 ng/ml with a mean correlation coefficient of 0.999. Quantification was achieved using peak area of compounds interpolated from the standard curve. Optimization: Extraction recoveries and feasibility were estimated comparing control FFPE brain tissue sections spiked with 5 *μ*l of 50 ng/ml of GDC-0152 and control sections spiked with just the solvent. Detection of GDC-0152 in treated FFPE mouse brain sections. The limit of detection corresponds to a signal to noise (S/N) ratio of ⩾3. The result obtained shows a detection of GDC-0152 in treated brains compared with control

As we showed that GBM cells were sensitive to GDC-0152 treatment, we decided to evaluate its antitumoral effect *in vivo*. A total of 21 mice were intracranially grafted with U87MG cells stably expressing near-infrared fluorescent protein (iRFP) in order to follow tumor growth *in vivo* as previously described.^12^ At 1 week after U87MG-iRFP cell grafts, mice were treated either with vehicle (dimethyl sulfoxide (DMSO), *n*=7) or with 10 mg/kg (*n*=7) or 20 mg/kg (*n*=7) of GDC-0152. Treatment started 1 week after cell injection and was performed once a week followed directly by tumor imaging. Treatment significantly increased mice survival in a dose-dependent manner (*P*=0.01; Figure 4a), postponed tumor formation and slowed down tumor growth (Figure 4b). Treatment was stopped when all the DMSO-treated mice were killed (60 days post injection) in order to test GDC-0152 long-term effect on the remaining mice. At weeks after the end of the treatment, only 1 out of 5 tumor-free mice developed a tumor in the ‘20 mg/kg' group, suggesting a long-lasting efficiency of the treatment (Figure 4). All the remaining GDC-0152-treated mice were killed 3 weeks after the end of the treatment. Histological analyses were performed on brains to check for tumor formation. Hematoxylin and eosin colorations confirmed imaging results. All DMSO-treated brains showed a tumor, whereas tumors were present in 5 out of 7 and 3 out of 7 of ‘10 mg/kg' and ‘20 mg/kg' groups, respectively, of GDC-0152-treated brains. Differentiation and cell death showed respectively by glial fibrillary acidic protein (GFAP; 20 mg/kg, *P*=0.015) and cleaved caspase-3 (10 mg/kg, *P*=0.03; 20 mg/kg, *P*=0.028) staining assays were increased in treated brains (Figures 5a and b). Furthermore, cell proliferation was reduced in GDC-0152-treated tumors as shown by a decrease in Ki67 staining (20 mg/kg, *P*=0.03; Figures 5a and b). Importantly, ultrahigh performance liquid chromatography–selected reaction monitoring (UPLC-SRM) analysis clearly confirmed that GDC-0152 was able to cross the blood–brain barrier and to properly diffuse into the brain to target orthotopic GBM xenograft (Figure 6). Moreover, the average of mice weight was identical in treated and untreated animals (data not shown) and histological analysis of kidney, liver, lung, heart and brain did not demonstrate any difference among treated and untreated animals (data not shown). Taken together, these data showed that the GDC-0152 was not toxic at the concentrations used.

These results revealed that GDC-0152 treatment improved xenografted mice survival and slowed down GBM tumor growth.

## Discussion

In this study, we highlighted the expression of cIAP1, cIAP2, XIAP and ML-IAP in an extensive cohort of human GBM. Moreover, we showed for the first time that ML-IAP was an independent prognostic marker in these tumors. Finally, our work also underscored the major role of IAPs in target therapy design as we showed the efficiency of SMAC mimetic GDC-0152 on GBM cell lines and GBM xenograft mice model.

Immunohistochemical detection of four IAPs in GBM showed that all cases expressed more than one IAP. Of particular interest was the expression of ML-IAP and high level was predictive of a worse prognosis. A previous study showed that ML-IAP was upregulated in GBM cell lines upon hypoxia leading to radio- and chemo-resistance.^13^ This previous work introduced ML-IAP as an actor of treatment resistance in GBM but the authors did not perform any correlation of ML-IAP expression with patient outcome. Because the standard of care for patients presenting a primary GBM is Stupp protocol,^2^ it is possible that the worse prognosis for patients with high ML-IAP level relies on *in vivo* radio- and chemo-resistance. Other studies have shown that ML-IAP expression was often associated with a bad prognosis in melanomas.^14^ In bladder cancer, ML-IAP expression was associated with early relapses^15^ and no correlation with patient outcome was found in other cancers.^16, 17, 18, 19, 20^ These results demonstrate the clinical relevance of using ML-IAP antagonist in GBM treatment.

In this study, we showed that GDC-0152, a SMAC mimetic used in monotherapy, demonstrated antitumoral effect in mouse orthotopic GBM xenograft model. Previous *in vitro* studies conducted on GBM showed that SMAC mimetics used in cotreatment sensitized GBM cells to temozolomide or *γ*-irradiations.^21, 22^ A recent work described a reduction in U87MG tumor growth in an orthotopic mouse model when combined with Drozitumab, a TRAIL-R2 (Tumor-necrosis-factor-Related Apoptosis-Inducing Ligand Receptor 2).^23^ However, effect on survival of these combination treatments or SMAC mimetic alone was not evaluated in these studies. Moreover, the authors used the bivalent SMAC mimetic BV6 described to antagonize cIAP1, cIAP2 and XIAP. Because we clearly demonstrated the clinical relevance of ML-IAP expression in GBM, we chose in this study to analyze the effect of the monovalent SMAC mimetic GDC-0152 that is described to antagonize not only cIAP1, cIAP2, XIAP and also ML-IAP.

The *in vitro* studies in other cancers showed that GDC-0152 effect inhibited PI3K/Akt. In human leukemia cells, GDC-0152 downregulated cIAP1, cIAP2 and XIAP proteins and induced apoptosis through caspase-9 and -3 activation and inhibition of PI3K/Akt pathway.^24^ In human osteosarcoma, GDC-0152 attenuated the metastasis properties of the SaOS2 cell line *via* PI3K/Akt inhibition.^25^ We showed here that GDC-0152 increased apoptosis and differentiation in all GBM cell lines. This effect was also observed in xenografts model. However, which signaling pathway is involved needs to be investigated.

Besides their anti-apoptotic function, IAPs have a broader role in tumorigenesis. We previously reported non-apoptotic functions of SMAC mimetics in GBM migration and GBM stem cell differentiation.^26^ Here, well-circumscribed tumors were obtained in DMSO control as well as in GDC-0152-treated mice showing no difference on cell migration/invasion upon GDC-0152 treatment. We previously reported that SMAC mimetic BV6 was able to reduce GBM stem-like cell properties *in vitro* and *in vivo*. After *in vitro* BV6 treatment, GBM stem-like cells were grafted orthotopically and lost their tumorigenic potential.^26^ We could not learn from this study whether GDC-0152 in monotherapy triggered GBM stem-like cells *in vivo.* It was obvious however that the two GBM cancer stem cell lines GBM6 and GBM9 were more resistant to GDC-0152 treatment *in vitro* than U87MG and GL261 cell lines.

Beyond effects in GBM cells, whether GDC-0152 would also activate apoptotic and non-apoptotic effects on other cellular compartments such as microenvironment remains to be determined.

SMAC mimetics are not tested yet in clinical trials for GBM and the present work underlines the need of such investigations. As SMAC mimetics are already under clinical investigations in other cancers, translation into clinic would be facilitated and attractive. As we demonstrated that ML-IAP is a particular attractive target, development of more specific ML-IAP antagonist would therefore be relevant.

## Materials and Methods

### Human glioblastoma samples

#### Local GBM cohort

GBM tumor specimens were obtained according to a protocol approved by the local institutional review board and ethics committee (2014-A00585–42) and conducted according to national regulations. All the patients provided written informed consent. Patient characteristics are summarized in Table 1. GBM formalin-fixed, paraffin-embedded (FFPE) samples provided by the AP-HM tumor bank (authorization AC-2013–1786) were pooled on several tissue-microarrays (TMAs) for high-throughput screening. Areas of viable and representative tumor following review of all blocks were marked by a pathologist (DF-B) before inclusion into the TMA (3 × 0.6 mm cores for each tumor). The first TMA cohort consisted of 58 patients with newly diagnosed *IDH1/2* wild-type GBM for whom clinical data were available.

In view of the results observed in the first cohort, we identified a second independent TMA cohort of 43 patients with *IDH1/2* wild-type GBM with available clinical data (Table 1).

### Immunohistochemistry and immunostaining quantification

After steam-heat-induced antigen retrieval, 5 *μ*m sections of FFPE samples were tested for the presence of cIAP1 (Rabbit polyclonal IgG AF8181, R&D Systems, Wiesbaden, Germany), cIAP2 (Mouse IgG AF8171, R&D Systems), XIAP (Mouse IgG1, clone 48, BD Biosciences, Franklin Lake, NJ, USA), ML-IAP (Mouse IgG1, IMG-347A, Imegenex, Cambridge, UK), GFAP (EP672Y, Ventana Medical Systems, Illkirch, France), Ki67 (30-9, Ventana Medical Systems) and caspase-3 (C92605, BD Biosciences). A Benchmark Ventana autostainer (Ventana Medical Systems) was used for detection, and slides were simultaneously immunostained in order to avoid intermanipulation variability. For negative controls, irrelevant antibodies with identical isotypes were used. Slides were then scanned (Nanozoomer 2.0-HT, Hamamatsu Photonics SARL France, Massy, France) and images processed in NDP.view2 software (Hamamatsu).

Based on immunostaining results, ML-IAP positivity was determined as percentage of positive cells and cIAP1, cIAP2 and XIAP were quantified as positive or negative staining. The percentage of GFAP-, Ki67- or cleaved-caspase-3-positive cells was determined as previously described.^26^

### Cell lines and reagents

GBM6 and GBM9 GBM stem cell lines were isolated in the laboratory from two different human GBM tumor samples and exhibited features reminiscent of the clinical characteristics of the original tumors respectively.^27^ These cells were grown as floating spheres in serum-free medium supplemented with epidermal growth factor (EGF) and basic fibroblast growth factor (bFGF) as previously described.^28^ For GDC-0152 treatment experiments, cells were grown in the same medium on 10 *μ*g/ml poly-DL-ornithin in order to allow cells to attach to the plastic without differentiating. Human GBM cell line U87MG (American Type Culture Collection, Rockville, MD, USA) and murine GBM cell line GL261 (gift from F Debarbieux, Aix-Marseille University, France) were cultured as monolayers respectively in Dulbecco's modified Eagle's medium (DMEM; Life Technologies, Saint Aubin, France) and RPMI (Life Technologies) supplemented with 10% fetal calf serum, 50 U/ml penicillin and 50 *μ*g/ml streptomycin. All the cell lines were grown at 37 °C in a humidified atmosphere of 5% CO_2_ and 95% air.

Monovalent SMAC mimetic GDC-0152 (Selleckchem, Houston, TX, USA) was dissolved at 30 mM for *in vitro* studies and 50 mM for *in vivo* studies in DMSO (Sigma-Aldrich, Saint-Quentin Fallavier, France) and stored at −80 °C until use.

### Cell viability assay

Effect of GDC-0152 on U87MG, GL261, GBM6 and GBM9 cell line viability was evaluated by assessing cell metabolic capacity using the MTT method (3-(4,5-dimethylthiazol-2yl)-diphenyl tetrazolium bromide; Sigma-Aldrich). GBM6 and GBM9 were seeded in previously poly-DL-ornithin-coated 96-well plates (10 000, 8000 and 6000 cells per well for 24, 48 and 72 h of treatment, respectively). U87MG and GL261 were seeded in 96-well plates (8000, 6500 and 5000 cells per well). After 24 h, cells were treated with serial concentrations of GDC-0152 (0.01, 0.05, 0.1, 0.5, 1, 5, 10 and 20 *μ*M) in 200 *μ*l of cell-specific media per well. After treatment, 20 *μ*l of MTT reagent was added to each well and plates were incubated for 4 h at 37 °C. The reduced formazan was dissolved in 200 *μ*l of DMSO and absorbance was measured at 562 nm with an Elx800 microplate reader (Bio-Tek, Colmar, France) and data were analyzed with Gen5 1.09 software (Bio-Tek).

### DNA fragmentation

Apoptosis was determined by flow cytometric analysis (FACSCalibur, BD Biosciences) of DNA fragmentation of propidium iodide-stained nuclei as described previously.^29, 30, 31^ U87MG and GL261 cell lines were seeded in 12-well plate (40 000 and 60 000 per well respectively), and 24 h after seeding, cells were treated with GDC-0152 for 72 h. GBM6 and GBM9 were seeded in previously poly-DL-ornithin-coated 12-well plate (25 000 per well) for 8 days of GDC-0152 treatment. Cells were fixed and permeabilized with 70% ethanol. The cells were then centrifuged, washed in phosphate-buffered saline (PBS) and resuspended in PBS supplemented with RNAse A (50 *μ*g/ml) and propidium iodide (40 *μ*g/ml). Cells were incubated in the dark at room temperature for 15 min and rapidly analyzed. Results were harvested with the CellQuest Pro Software (BD Biosciences) and analyzed using FlowJo software (Ashland, OR, USA).

### Protein extraction and western blotting

Proteins were extracted with tris(hydroxymethyl)-aminomethane hydrochloric acid (Tris-HCl) pH 7.4 (50 nM), NaCl ([250 mM), EDTA (5 mM), dithiothreitol (DTT; 1 nM), 1% Triton X-100, 0.1% SDS (1 M), 0.5% sodium deoxycholate, 1% Nonidet P-40 (NP-40) and Complete 1 × (Roche Applied Science, Meylan, France). After two sonications, cells were maintained 20 min on ice and centrifuged for 10 min at 12 000 revolutions per min (r.p.m.). Protein concentration was assayed using bicinchoninic acid (MicroBCA kit, Pierce, Rockford, IL, USA). Then, 50 *μ*g proteins per lane were separated by 12% sodium dodecylsulfate–polyacrylamide gel electrophoresis (SDS-PAGE) and transferred onto nitrocellulose membrane (iBlot gel transfer, Life Technologies). After blocking for 2 h in PBS supplemented with 5% skimmed milk, immunodetection was performed using anti-cIAP1 (1 : 1000, AF8181, R&D Systems), anti-cIAP2 (1 : 1000, clone E40, Epitomics, Burlingame, CA, USA), anti-XIAP (1 : 1000, clone 28, BD Biosciences), anti-ML-IAP (1 : 1000, clone 88C570, Imgenex, San Diego, CA, USA) and anti-*β*-actin (1 : 5000, Sigma-Aldrich) in PBS supplemented with 5% bovine serum albumin (BSA) and 0.1% Tween-20 (Sigma-Aldrich), followed by horseradish peroxidase-conjugated goat anti-mouse IgG (Santa Cruz Biotechnology, Santa Cruz, CA, USA) or rabbit anti-goat IgG (Dako France, Ulis, France). ECL (Bio-Rad, Marne-la-Coquette, France) was used for detection.

### iRFP infection

The HIV-derived lentiviral vector encompassing the iRFP complementary DNA (cDNA), viral particle preparation and cell transduction were performed essentially as described.^12^ Briefly, the iRFP construct was made by replacing the enhanced green fluorescent protein (EGFP) gene in the lentiviral vector pRRLSIN.cPPT.PGK-GFP.WPRE^32^ (referred to as pRRL) by the iRFP cDNA originating from the pShuttle-CMV-iRFP plasmid (a gift of Dr. Verkhusha, Addgene plasmid 31856, Teddington, UK). In order to easily monitor transfection and transduction events, we chose the simultaneous expression of iRFP and the red fluorescent protein mCherry (Clonetech, Mountain View, CA, USA) using the internal ribosomal entry sequence (IRES) system. For this purpose the IRES-mCherry DNA cassette was cut from pcDNA3/IFP1.4 (kindly provided by Dr. Tsien, Scripps Clinic, La Jolla, CA, USA) with *Xho*I and *BsrG*I and cloned at the 3′ end of the iRFP sequence between *Sal*I and *Acc*651 sites, giving rise to pRRL/iRFP/IRES-mCherry, thus driving the expression of a single bicistronic mRNA encoding both IRFP and mCherry. Lentiviral particles preparation and infection of the human GBM U87MG cells with viral particles were performed according to Mathieu *et al.*^33^

### Intracranial injections

All experimental procedures using animals were carried out according to a protocol approved by institutional review board and the French ethical committee. This project received the authorization number 02313.01. A total of 100 000 U87MG-iRFP cells were stereotactically injected in the corpus callosum (+1 mm anterior to bregma, −1 mm lateral and −2 mm in deep of the cortex surface) of 21 6-week-old athymic *nude* mice as previously described.^28^ Animals were observed until they fully recovered. The body weight and clinical status of mice were recorded every 2 days. Mice were killed when they exhibited >20% reduction from initial body weight or significant neurological deficit. Mice were weekly intravenously treated with GDC-0152 (10 and 20 mg/kg) or with vehicle (highest volume of DMSO). Treatment started 1 week after cell graft and was stopped when all the DMSO-treated mice were killed (60 days post graft). These concentrations were previously reported to be nontoxic in dogs and rats,^10, 34, 35^ but, to our knowledge, no data regarding toxicity in mice were available in literature. Directly after their killing, brains were extracted, fixed in formalin and paraffin embedded according to standard procedures. In addition, we also collected liver, kidney, lung, heart and brain from 3 control mice and 3 mice treated with 20 mg/kg GDC-0152 to check cell toxicity on selected organs. Hematoxylin and eosin colorations were performed on 5 *μ*m sections of FFPE brains in order to validate tumor development.

### UPLC-SRM

Two pools of mice grafted with U87MG-iRFP and demonstrating brain tumors were used. Twenty sections from FFPE tissue blocks of GDC-0152 treated mice and of DMSO control mice were deparaffinized by incubating with 1 ml of xylen for 10 min at room temperature followed by centrifugation at 20 000 × *g* for 5 min. The supernatant was collected and transferred to a clean Eppendorf tube. The extract was dried in a concentrator. The dry extracts was then suspended in 20 *μ*l of acetonitrile/0.1% formic acid (50 : 50).

UPLC-SRM was performed on a Nexera LC system (Shimadzu Corporation, Tokyo, Japan) coupled to a Triple quadripole 8040 mass spectrometry system (Shimadzu Corporation). Separation of GDC-0152 was carried out using a Kinetex XB-C18 column (150 × 2.1 mm), 2.6 *μ*m particle size, Phenomenex with a column temperature of 30 °C. Elution was obtained by a linear gradient from 5 to 50% of acetonitrile in 0.1% formic acid over 6 min at a flow rate of 0.4 ml/min. Next, 5 *μ*l of the extract was injected on the UPLC-SRM. After automatic optimization of collision energies for GDC-0152, the transitions were set to 499.2–>275 and 499.2–>70.1. Data acquisition and analysis were performed using Labsolution v5.6 software from Shimadzu Corporation.

### *In vivo* imaging and analysis

Image acquisition of U87MG-iRFP-injected mice was taken directly after treatment with a Pearl Impulse Small Animal Imaging System (Li-Cor Biosciences GmbH, Bad Hamburg, Germany) and the analysis was performed using Image Studio Lite software (Li-Cor Biosciences GmbH) as previously described.^12^ Images of all mice were linked in order to apply the same fluorescence intensity settings. Data were then normalized to the background and to the lowest fluorescence intensity, and are presented as arbitrary units (a.u.) according to Filonov *et al.*^36^

### Statistical analysis

Categorical variables were presented as frequencies and percentages and continuous variables as median and range. OS was defined to be time from the date of surgery to death, censored at the date of last contact. PFS was defined to be time from the date of surgery to documented progression or death, censored at the date of the last documented disease evaluation. The Kaplan–Meier method was used to estimate survival distributions. Log-rank tests were used for univariate comparisons. Cox proportional hazards models were used for continuous and multivariate analyses and to estimate HRs in survival regression model. Multivariate analysis included all variables (gender, age, KPS and type of surgery) with a *P*-value of <0.05. Mann–Whitney *U*-test was used to compare quantitative values; qualitative values were compared using the *χ*^2^ test or Fisher's exact test. For survival analyses, subjects were divided into two groups based on their optimal cutoff determined by the ROC analysis for the quantitative variable (ML-IAP) or into positive and negative groups for the qualitative variables (cIAP1, cIAP2 and XIAP). All the tests were two sided and *P*-values of <0.05 were considered significant for each statistical analysis.

Statistical analyses were conducted using the statistical package SPSS software v.22 (SPSS Inc., Chicago, IL, USA).

## Figures and Tables

**Table 1:** Patient characteristics

**Characteristics**	**TMA 1,** ***N*****=58**	**%**	**TMA 2,** ***N*****=43**	**%**
Median age (range)	61.7 (20.5–81.9)		57 (21.1–78.7)	
*KPS*				
<70	14/58	24.14	Unknown	
⩾70	44/58	75.86	Unknown	
*Type of surgery*				
Gross total resection	37/57	63.8	16/42	61.9
Other (partial excision, biopsy)	20/57	36.2	26/42	38.1
*MGMT promoter*				
Methylated	19/53	36	5/17	29.4
Unmethylated	34/53	64	12/17	70.6
*Markers*				
				
*cIAP1*				
Protein expression (positive/negative, range %)	31/25 (0–60)	55.3/44.7	19/19 (0–30)	50/50
* cIAP2*				
Protein expression (positive/negative, range %)	41/11 (0–10)	78.8/21.2	6/31 (0–15)	16.2/83.8
*XIAP*				
Protein expression (positive/negative, range %)	19/39 (0–90)	32.7/67.3	25/14 (0–70)	64.1/35.9
				
*ML-IAP*				
Protein expression (median, range %)	30 (0–100)		15 (0–80)	

## Abbreviations

a.u.: arbitrary unit
bFGF: basic fibroblast growth factor
BIR: baculovirus IAP repeat
BSA: bovine serum albumin
cIAP1/2: cellular inhibitor of apoptosis protein-1/2
cDNA: complementary DNA
DMEM: Dulbecco's modified Eagle's medium
DMSO: dimethyl sulfoxide
DTT: dithiothreitol
ECL: enhanced chemiluminescence
EGF: epidermal growth factor
EGFP: enhanced green fluorescent protein
FFPE: formalin-fixed, paraffin-embedded
GBM: glioblastoma
GFAP: glial fibrillary acidic protein
HCl: hydrochloric acid
HR: hazard ratio
IAP: inhibitor of apoptosis protein
IgG: immunoglobulin G
iRFP: near-infrared fluorescent protein
ML-IAP: melanoma inhibitor of apoptosis protein
MTT: bromure de 3-(4,5-dimethylthiazol-2-yl)-2,5-diphenyl tetrazolium
NP-40: Nonidet P-40
OS: overall survival
PAGE: polyacrylamide gel electrophoresis
PBS: phosphate-buffered saline
PFS: progression-free survival
RING: real interesting new gene
ROC: receiver operating characteristic
r.p.m.: revolutions per min
SDS: sodium dodecylsulfate
SRM: selected reaction monitoring
Smac: second mitochondria derived activator of caspases
TMA: tissue microarray
TRAIL-R2: necrosis-factor-related apoptosis-inducing ligand receptor 2
Tris: tris(hydroxymethyl)-aminomethane
UPLC: ultrahigh performance liquid chromatography
XIAP: X-linked inhibitor of apoptosis protein
