# Supplementary material for: Inhibitor of apoptosis protein expression in glioblastomas and their in vitro and in vivo targeting by SMAC mimetic GDC-0152
Source: Cell Death Dis. 2016 Aug 4;7(8):e2325–. doi: 10.1038/cddis.2016.214 (PMC5108315; doi:10.1038/cddis.2016.214)
Supplement: Supplementary Information [file cddis2016214x1.docx]

**Inhibitor of apoptosis proteins expression in glioblastomas and their *in vitro* and *in vivo* targeting by SMAC mimetic GDC-0152**

**Aurélie Tchoghandjian^1,#^, Aurélie Soubéran^1,#^, Emeline Tabouret^1,2^, Carole Colin^1^, Emilie Denicolaï^1^, Carine Jiguet-Jiglaire^1^, Assou El-Battari^1^, Claude Villard^1^, Nathalie Baeza-Kallee^1^ and Dominique Figarella-Branger^1,3,*^**

**Supplementary Figure S1. ML-IAP ROC curve and XIAP survival curves.**

**(A)** ML-IAP ROC curve determined an area under the curve (AUC) of 0.7.

**(B)** XIAP protein expression association with progression free survival (PFS) and overall survival (OS) in cohort 1. Positive XIAP expression was associated with a poor OS.

**Supplementary Figure S2. Univariate and multivariate analyses of progression-free survival and overall survival of cohort 1 and 2.**

PFS: progression-free survival; OS: overall survival; NA: not analyzed. 95% confidence interval is given in parenthesis.

**Supplementary Figure S3. Survival analyses in the pooled cohorts 1 and 2.**

PFS: progression-free survival. OS: overall survival. HR: hazard ratio. 95% confidence interval is given in parenthesis.

**Supplementary Figure S4. Cell viability upon GDC-0152 treatment.**

U87MG, GL261 and GBM6 and GBM9 cell lines were treated with increasing concentrations of GDC-0152 for the indicated times. Cell viability was measured by MTT assay and was expressed as fold increase of untreated controls. GBM6 and GBM9 were grown in monolayer. GDC-0152 affected U87MG and GL261 cell viability in time- and dose-dependent manner. Viability of GBM6 and GBM9 cell lines was barely affected in these conditions. Mean + s.e.m is shown. Three independent experiments were performed for U87MG and GL261 cell lines, four for GBM6 cell line and five for GBM9 cell line. Each experiment was performed in triplicate. **p*<0.05.
