# Supplementary figures and images for: Inhibitor of apoptosis protein expression in glioblastomas and their in vitro and in vivo targeting by SMAC mimetic GDC-0152
Source: Cell Death Dis. 2016 Aug 4;7(8):e2325–. doi: 10.1038/cddis.2016.214 (PMC5108315; doi:10.1038/cddis.2016.214)

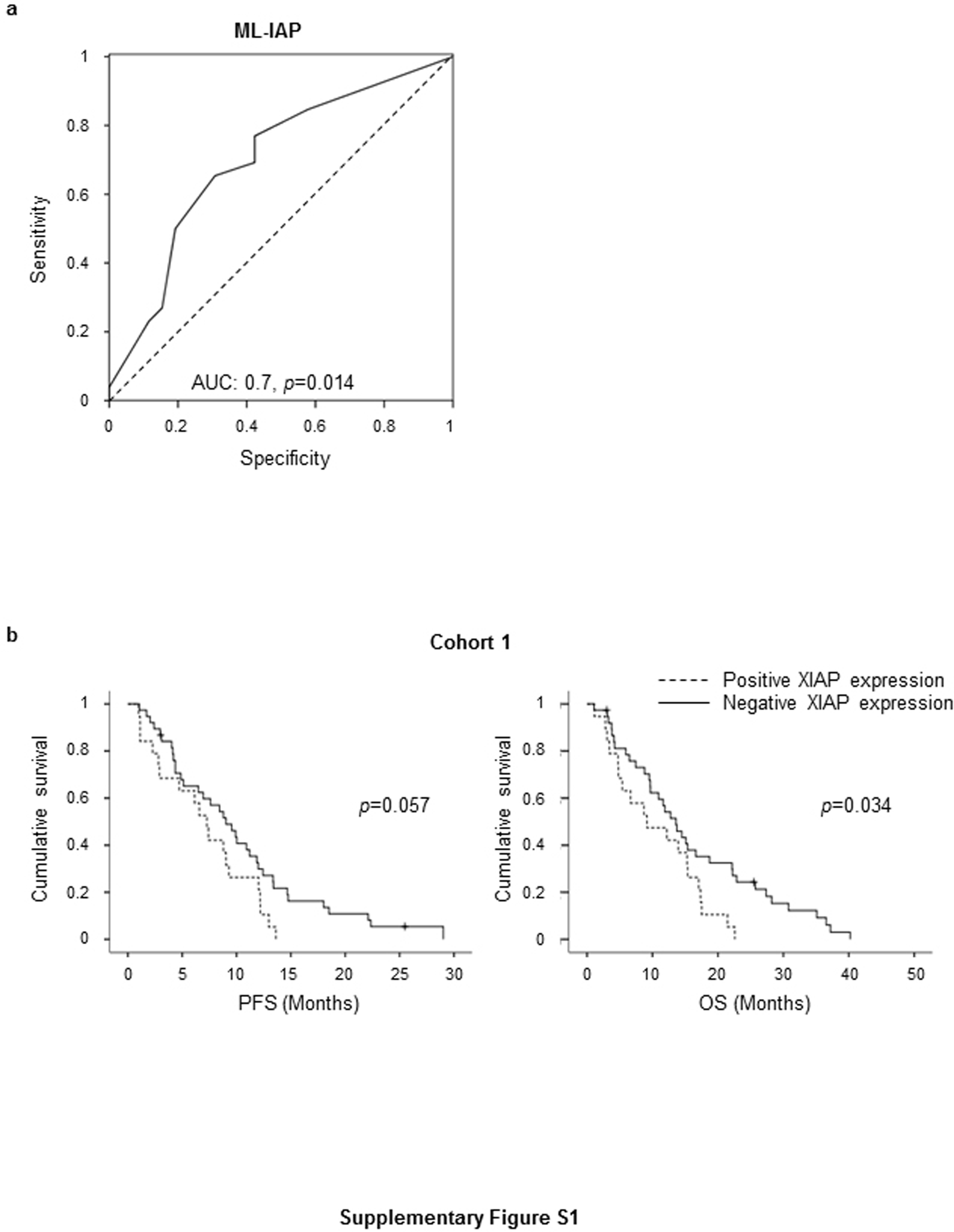

Supplement: Supplementary Figure S1 [file cddis2016214x2.tif]

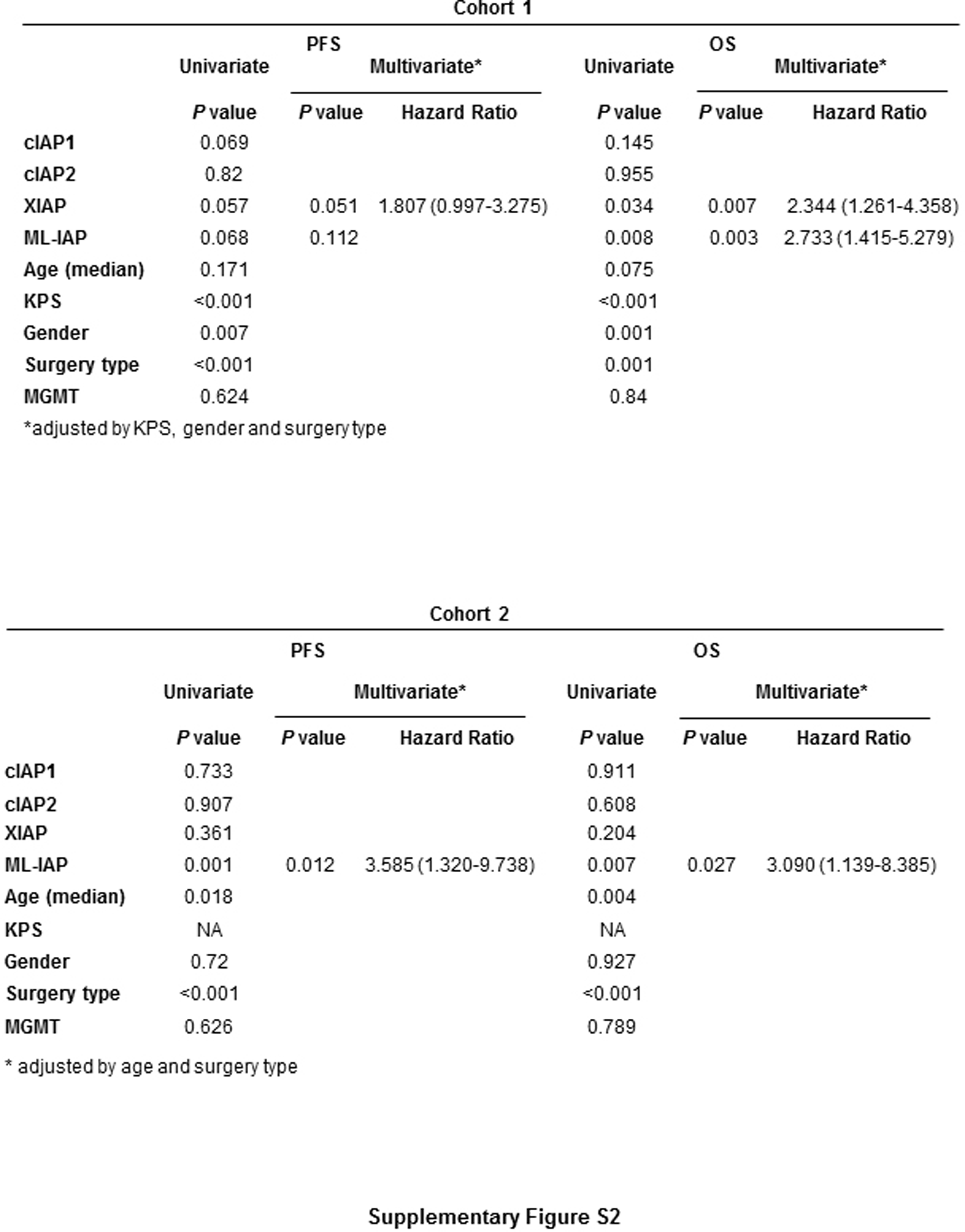

Supplement: Supplementary Figure S2 [file cddis2016214x3.tif]

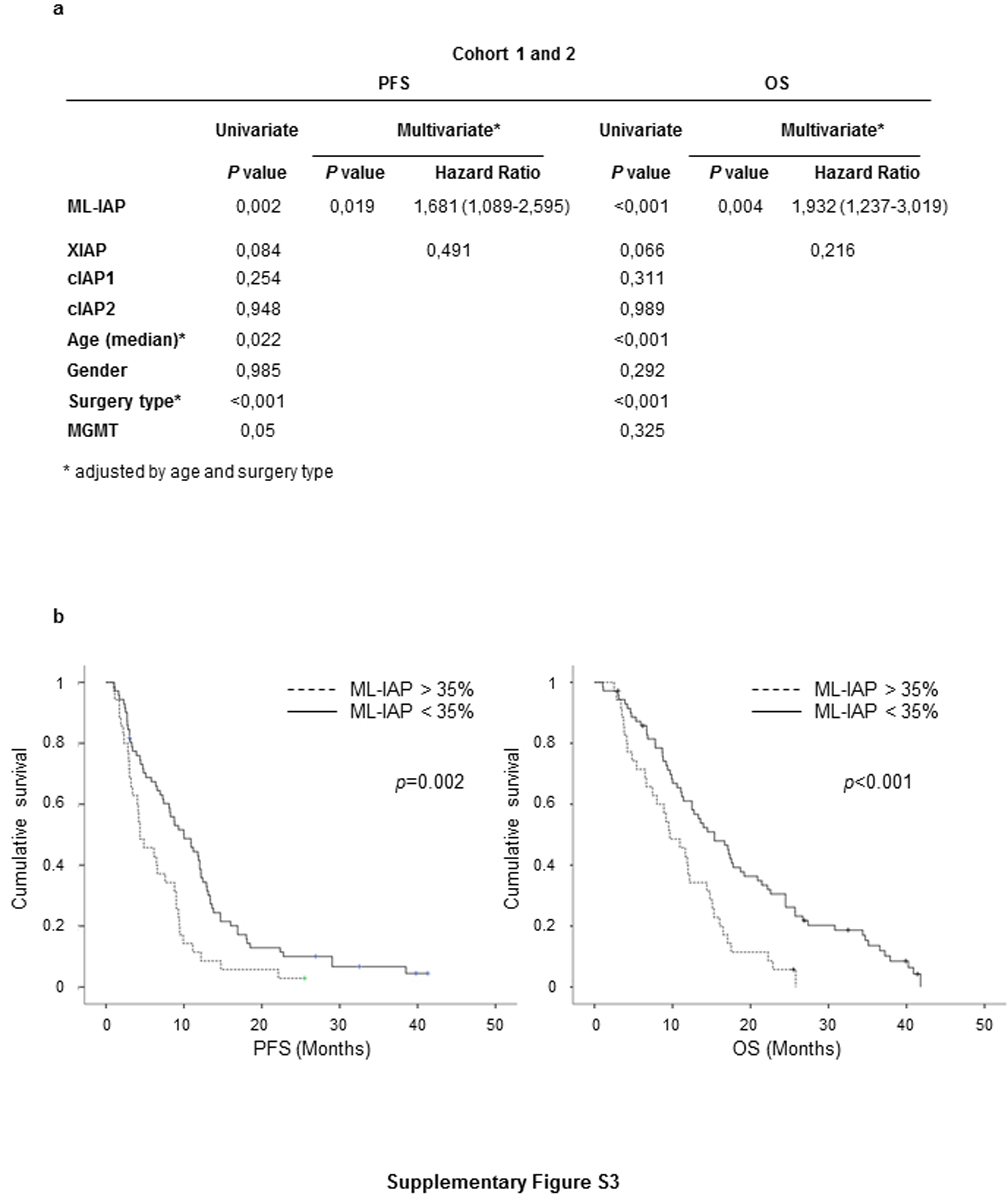

Supplement: Supplementary Figure S3 [file cddis2016214x4.tif]

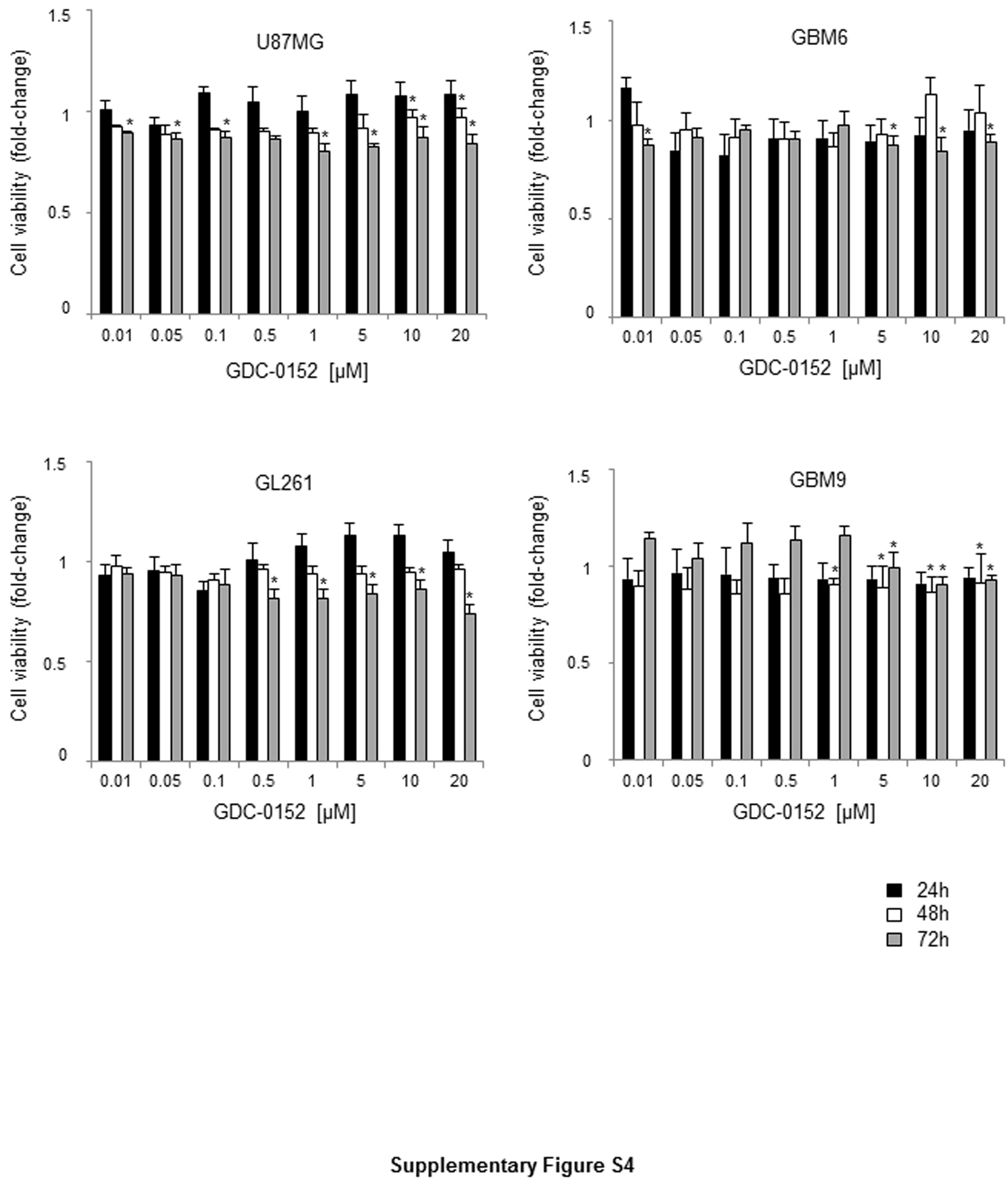

Supplement: Supplementary Figure S4 [file cddis2016214x5.tif]
